# Coding-complete genome sequences of two bovine viral diarrhea virus 1a isolates from Uruguay

**DOI:** 10.1128/mra.00917-23

**Published:** 2024-02-15

**Authors:** L. Maya, Y. Panzera, R. Pérez, A. Marandino, R. Colina

**Affiliations:** 1 Laboratorio de Virología Molecular, Departamento de Ciencias Biológicas, CENUR Litoral Norte-Sede Salto, Universidad de la República, Gral. Rivera, Uruguay; 2 Sección Genética Evolutiva, Departamento de Biología Animal, Instituto de Biología, Facultad de Ciencias, Universidad de la República, Montevideo, Uruguay; Katholieke Universiteit Leuven, Leuven, Belgium

**Keywords:** bovine, bovine viral diarrhea virus, coding-complete, isolate

## Abstract

Bovine viral diarrhea virus (BVDV) is an economically relevant pathogen affecting cattle production and reproduction worldwide. We report the coding-complete sequences of two BVDV-1a subtype isolates, circulating in Uruguay.

## ANNOUNCEMENT

Bovine viral diarrhea virus (BVDV) is a pathogen of global economic importance affecting cattle production and reproduction (1). In Uruguay, BVDV causes reproductive failures, abortions, and other associated diseases affecting mucosal, respiratory, and enteric systems (2–4). BVDV belongs to the genus *Pestivirus* in the family *Flaviviridae*. It has a single-stranded positive-sense RNA genome of approximately 12.3 kb in length, with one open reading frame (ORF) that encodes a polyprotein of 3,898 amino acid residues flanked at both ends by untranslated regions (UTRs) (5).

There are three BVDV species, BVDV-1, BVDV-2, and HoBi-like pestivirus, recently renamed as *Pestivirus bovis*, *Pestivirus taur*i, and *Pestivirus brazilense* (6).

The BVDV-1a subtype is predominant in Uruguay and is genetically distant from the NADL vaccine strain, potentially causing vaccination failure (7). We report the coding-complete sequences of two BVDV-1a Uruguayan isolates obtained by Illumina sequencing Technology. 754UYAFA4/112015 and 2402UYSJ/2016 were isolated from serum samples of BVDV-symptomatic calves collected from dairy herds in 2015 and 2016, respectively. For virus isolation, Madin-Darby bovine kidney cells (MDBK) were used with minimum essential medium (MEM) supplemented with 10% horse serum and antibiotics (Penicillin, Streptomycin, and Amphotericin B). The plates were incubated at 37°C with 5% CO_2_ and examined daily for 3 days. Three consecutive blind passages were performed, and cell infection was determined by an antigen capture ELISA (IDEXX- Switzerland) because BVDV is a non-cytopathic virus. Infected cells were submitted to three freeze/thaw cycles to release viral particles and centrifuged at 3,000 × rpm for 3 min to clarify the supernatant. RNA was isolated using 1 mL of cultured supernatant using a Quick-RNA MiniPrep kit (Zymo Research, USA). The extracted RNA was reverse-transcribed using random hexamer primers using the Maxima H Minus Double-Stranded cDNA Synthesis kit (Thermo Fisher Scientific, USA). The cDNA was purified using AMPure XP (Benchman, USA), and 100 ng was subjected to Nextera DNA Flex Library Preparation kit (Illumina, USA). The libraries were sequenced on an Illumina MiniSeq platform using MiniSeq Mid Output Reagent Cartridge (paired-end 300 cycle). Adapter/quality trimming and filtering of raw data were performed with BBDuk (Geneious Prime 2023.0.1). For sample 754UYAFA4/112015 and 2402UYSJ/2016, 8,021 and 10,592 BVDV-specific clean reads were obtained which were mapped to BVDV-1a Oregon C24V strain (AF091605, the closest genome determined by the NIH BLAST tool) using Geneious algorithm of Geneious Prime 2023.0.1. The coding-complete sequences obtained had an average coverage of 78.3 and 102.7, respectively (Table 1).

**TABLE 1:** Sequencing metrics, and accession numbers for 754UYAFA4/112015 and 2402UYSJ/2016

	BVDV-specific reads	Avg. coverage (x)	GC content (%)	GenBank acc. number	SRA acc. number	Length of coding-complete sequences (nt)	ORF length (nt)
754UYAFA4/112015	8,021	78.3	45.9	OR620202	SRR26222711	12.279	11.694
2402UYSJ/2016	10,592	102.7	45.9	OR620203	SRR26223076	12.213	11.694

Table 1 summarizes the sequencing metrics and accession numbers of Uruguayan strains. Using Oregon C24V strain as reference, an ORF, of 11,697 nucleotides (3,898 amino acid residues), was identified on the forward strand of both coding-complete sequences.

Uruguayan strains were highly similar among them (nucleotide and amino acid identity 94.7% and 96.7%, respectively), and to Oregon C24V strain (nucleotide and amino acid identity 92% and 95%, respectively), and genetically distant to the NADL vaccine strain (nucleotide and amino acid identity were 88% and 92%, respectively). Before our report, there are neither Uruguayan BVDV-1a isolates nor coding-complete sequences; thus, our results adds valuable information.

## Data Availability

The coding-complete sequences of 754UYAFA4/112015 and 2402UYSJ/2016 strains were deposited to the GenBank database under accession numbers OR620202 and OR620203, and SRA SRR26222711 and SRR26223076, respectively (Table 1).

## References

[1] Houe H. 1999. Epidemiological features and economic importance of bovine virus diarrhoea virus (BVDV) infections. Vet Microbiol 64:89–107. doi:10.1016/s0378-1135(98)00262-4 10028165

[2] Maya L, Puentes R, Reolón E, Acuña P, Riet F, Rivero R, Cristina J, Colina R. 2016. Molecular diversity of bovine viral diarrhea virus in Uruguay. Arch Virol 161:529–535. doi:10.1007/s00705-015-2688-4 26597189

[3] Macías-Rioseco M, Silveira C, Fraga M, Casaux L, Cabrera A, Francia ME, Robello C, Maya L, Zarantonelli L, Suanes A, Colina R, Buschiazzo A, Giannitti F, Riet-Correa F. 2020. Causes of abortion in dairy cows in Uruguay. Pesq. Vet. Bras 40:325–332. doi:10.1590/1678-5150-pvb-6550

[4] da Silva Silveira C, Maya L, Casaux ML, Schild C, Caffarena D, Aráoz V, da Costa RA, Macías-Rioseco M, Perdomo Y, Castells M, Colina R, Fraga M, Riet-Correa F, Giannitti F. 2020. Diseases associated with bovine viral diarrhea virus subtypes 1A and 2B in beef and dairy cattle in Uruguay. Braz J Microbiol 51:357–368. doi:10.1007/s42770-019-00170-7 31650465 PMC7058746

[5] Meyers G, Thiel HJ. 1996. Molecular characterization of pestiviruses. Adv Virus Res 47:53–118. doi:10.1016/s0065-3527(08)60734-4 8895831

[6] International committee on taxonomy of viruses ICTV. Available from: https:// talk.ictvonline.org/

[7] Maya L, Macías-Rioseco M, Silveira C, Giannitti F, Castells M, Salvo M, Rivero R, Cristina J, Gianneechini E, Puentes R, Flores EF, Riet-Correa F, Colina R. 2020. An extensive field study reveals the circulation of new genetic variants of subtype 1A of bovine viral diarrhea virus in Uruguay. Arch Virol 165:145–156. doi:10.1007/s00705-019-04446-z 31745717 PMC7222985

